# Thermal performance of Casson hybrid nanofluid over expanding/contracting wedges with joule dissipation: a Falkner–Skan problem

**DOI:** 10.1038/s41598-026-58305-4

**Published:** 2026-06-17

**Authors:** Subhajit Panda, Rupa Baithalu, S. R. Mishra, Laila A. AL-Essa, Anwar Saeed, Gabriella Bognár

**Affiliations:** 1 Centre for Data Science, Department of Computer Science and Engineering, Siksha ‘O’ Anusandhan Deemed to be University, Bhubaneswar, Odisha 751030 India; 2 Department of Mathematics, Siksha ‘O’ Anusandhan Deemed to be University, Bhubaneswar, Odisha 751030 India; 3 Department of Mathematical Sciences, College of Science, Princess Nourah bint Abdulrahman University, P.O.Box 84428, 11671 Riyadh, Saudi Arabia; 4 Department of Computer Engineering, Biruni University, 34010 Istanbul, Turkey; 5 Institute of Machine and Product Design, University of Miskolc, Miskolc-Egyetemváros, 3515 Hungary

**Keywords:** Casson hybrid nanofluid, Falkner–Skan model, Thermal radiation, Joule dissipation, Numerical technique, Engineering, Mathematics and computing, Nanoscience and technology, Physics

## Abstract

The present investigation explores the heat-transfer attributes and fluid-flow properties of Casson hybrid nanofluid comprising of Cobalt and gold nanoparticles in the base liquid paraffin over an expanding/contracting surface using the Falkner–Skan model. The combination of the applied magnetic field, thermal radiation, and magnetic dissipation improves flow properties and thermal flow attributes. The augmented thermal efficacy of the hybrid nanofluid synergistically amplifies the heat transfer performance of the Casson fluid framework., which accounts for the non-Newtonian behaviour of the fluid, including radiation that augments the heat transfer near the wedge surface, whereas Joule dissipation introduces heat generation. The mathematical model presented for the proposed assumptions is transformed into dimensionless form utilizing transformation rules and then solved numerically, adopting the shooting method and integrating the Runge–Kutta fourth-order technique. The significant characteristics of several factors are presented graphically and elaborated briefly. It is noted that the non-Newtonian rheological characteristic of the fluid retards the thickness of the velocity bounding surface, and the enhanced Casson parameter favours augmenting the heat transfer rate. This enhancement is greater for the bi-hybrid nanofluid compared to the single nanofluid.

## Introduction

Casson hybrid nanofluids have been studied for their applications in biomedical systems (e.g., drug delivery, hyperthermia treatment), industrial cooling processes, and energy systems like solar collectors and heat exchangers. Their behaviour under varying flow regimes, including porous media, stretching/shrinking surfaces, and rotating geometries, has provided valuable insights into optimizing energy efficiency. Furthermore, the inclusion of nanoparticles such as graphene oxide, titanium dioxide, silver, or alumina enhances thermal and electrical conductivity, making these fluids ideal for advanced applications. Understanding the flow behaviour and energy efficiency of Casson hybrid nanofluids is essential for optimizing their properties for targeted applications. The analysis becomes more intricate due to the interpretation of magnetisation, radiation, buoyancy, and chemical reactions on these fluids. Abbas et al.^1^ investigated the couple stress tensor in a Casson polar hybridised fluid transporting over an exponentially curved, elastic material. Additionally, the effects of radiant heat and Ohmic heating are investigated, as is the function of Lorentz forces in controlling fluid flow close to the extending surface. Moreover, a thorough comparison of the “Yamada-Ota and Xue” hybrid nanofluid models is also involved in their analysis. Also, in a separate study, the characteristics of the Casson fluid model across a porous material were investigated by Tufail et al.^2^. Their research focused on an upward channel with mixed convection flow, incorporating the interpretation of radiative heat and slip situations along the channel walls. The Lie group method was deployed to transmute the nonlinear coupled PDE into nonlinear ODEs. Lastly, the outcomes showed that a higher Casson parameter lowers the concentration profile and increases the velocity profile. Tufail et al.^3^ addressed the interpretation of a Casson fluid across a permeable, inclined, vertically stretching sheet. Also, they emphasized the integration of MHD, viscous dissipation, magnetic heating, and reactive chemical species on different boundary layers. The governing models were mathematically solved in MATLAB with the bvp4c solver, and the results were presented as graphical and tabular representations. Saleem et al.^4^ observed the attributes of a non-viscous fluid model in the context of a radiative source, while another study by Saleem et al.^5^ explored the Casson fluid model via the “Lie scaling method” for two specific regulating factors. Das et al.^6^ explored the performance of a conducting dusty liquid described by the Casson fluid model, considering a thermally active plate with ramped motion and the influence of magnetism. Their study incorporated boundary conditions involving Newtonian heating, heat generation, and thermal radiation. To obtain an analytical solution, they applied the Laplace transform method. In another study, Das et al.^7^ discovered the rotational movement of a Casson fluid, emphasizing the effects of rotational buoyancy thermal plate. In a separate study, the flow of a Casson tri-hybridized material about a cylinder with a nonlinear velocity profile was observed by Paul et al.^8^. Nadeem et al.^9^ scrutinized the dynamics of a viscous nanofluid in a two-dimensional magnetohydrodynamic framework, emphasizing its flow along a coiled surface during mass extraction. Huang et al.^10^ examined the interpretation of radiating heat, Ohmic heating, and viscous dissipation on heat transportation efficiency and inertial resistance in a viscous nanofluid flowing over a curved bended material. Nadeem et al.^11^ used a hybrid modelling technique to investigate nanofluid movement along a curved surface. Gohar et al.^12^ inspected the inertial drag behaviour of a hybrid nanofluid on a curved surface that extends continuously.

Thermal radiation, Joule dissipation, and numerical techniques play vital roles in understanding and modelling heat transfer in various systems. Thermal radiation, a significant mode of thermal transportation, influences the temperature distribution and energy balance, especially at high temperatures or in vacuum environments. Joule dissipation, resulting from the conversion of electrical energy into heat due to the resistance in conductive materials, is crucial in the analysis of electrical and electronic systems. These methods allow for detailed predictions of heat transfer processes and enable the optimization of thermal regulation in industrial areas. Kumar et al.^13^ researched the thermal flow attributes of an MHD micro rotational fluid induced by surface stretching with a second-order velocity slip, incorporating nonlinear radiating energy and non-uniform heat sources and sinks. Prameela et al.^14^ explored the attribute of hydromagnetic Casson fluid via a vertically oscillating surface and reported a reduction in momentum pattern due to magnetohydrodynamic effects. Turkyilmazoglu^15^ scrutinized the magnetized movement of a non-Newtonian fluid over a shrinkable plate. Uddin et al.^16^ anticipated the consequence of heat transport on boundary layer MHD flow using particle swarm optimisation and four different nanoparticles. According to their findings, increasing heat absorption and suction parameters improves heat transfer. Similarly, Thumma et al.^17^ did a computational scrutiny on the influence of viscous dissipation and magnetic heating in a Jeffery nanofluid flowing over a horizontally elastic material. Finally, the results showed an excellent correlation with previous works, with the numerical solutions being validated through limiting cases. Additionally, it was observed that heat transportation was more pronounced for water-based nanofluids compared to others. In the boundary layer domain, skin friction exhibited fluctuating behaviour, especially in multi-diffusive MHD fluid scenarios, as noted by Patil et al.^18^. Magagula et al.^19^ addressed the transportation of a Casson nanofluid via a heated surface containing motile microbes. Kairi et al.^20^ spotlighted the MHD transportation of Casson nanofluid via an inclined elongating material, under the interpretation of motile microbes. Dhlamini et al.^21^ studied the movement of chemically reactive nanofluids influenced by bioconvection and Arrhenius kinetics. Izady et al.^22^ examined the flow pattern of Fe₂O₃–CuO hybrid nanofluid over a permeable stretching/shrinking wedge, building upon the classical Falkner–Skan problem. Yang et al.^23^ showed a CFD study on paraffin-based hybrid (Co–Au) and trihybrid (Co–Au–ZrO₂) nanofluids flowing via porous substrate, highlighting intricate flow patterns. Qamar et al.^24^ scrutinized Williamson fluid flow over a shrinking disk by incorporating the Cattaneo–Christov heat flux model and chemical reaction effects. Their findings demonstrated the substantial influence of relaxation phenomena and chemical reactions on thermal and concentration distributions. In another study, Qamar et al.^25^ numerically investigated convective heat transfer characteristics of hybrid nanofluids subjected to magnetic dipole effects and heat generation/absorption mechanisms. Their analysis revealed that magnetic and thermal source parameters significantly modify the thermal transport process. Khan et al.^26^ explored chemically reactive hybrid nanofluid flow involving cubic autocatalytic reactions and reported the existence of multiple solutions under specific flow conditions, highlighting the complexity of nonlinear transport phenomena. The flow behavior of Casson fluids containing hybrid nanoparticles has also received considerable attention. Manohar et al.^27^ analyzed Casson fluid flow through a porous microchannel and demonstrated that the incorporation of hybrid nanoparticles markedly improves heat transfer performance. Puneeth et al.^28^ studied three-dimensional mixed convection flow of a hybrid Casson nanofluid over a nonlinear stretching surface using a modified Buongiorno model. Wang et al.^29^ investigated the non-Darcian flow of radiative ternary hybrid Casson nanofluids over a moving rotary cone. Their computational study showed that thermal radiation and porous medium effects considerably enhance thermal transport characteristics. Wang et al.^30^ examined entropy generation and melting heat transfer in a Carreau fluid flowing over a Riga plate with variable thickness using the Keller–Box numerical method.

*Key aspect of the proposed study* Building on the previously discussed literature, this research seeks to investigate:

- Explore the behaviour of thermofluidic transport characteristic of Casson hybrid nanofluid integrating the impact of Cobalt and gold nanoparticles in paraffin.
- Investigate the role of conducting fluid over an expanding/contracting wedge surface for the Falkner–Skan model.
- Analyze the association of thermal radiation and magnetic dissipation i.e. Joule dissipation on the enhancement of fluid transport and thermal exchange processes.
- Present the interpretation of synergetic factors in designing advanced thermal management systems, particularly for improved aircraft safety.

*Novel approach of the study* The following few aspects of the proposed investigation shows its novel approach and these are;

- A novel investigation of Casson nanofluid containing the hybridization of Co and Au nanoparticles in paraffin through an expanding/contracting wedge.
- The combined impact magnetic field, thermal radiation, Joule dissipation are incorporated.
- Comparative assessment of hybrid and single nanofluid systems demonstrate the superior heat transfer capability of the proposed hybrid nanofluid.
- The influence of Casson parameter on the thermal enhancement is systematically quantified showing the role of non-Newtonian behavior.

*Research questions* Few of the research questions those are vital in the progress of the present investigation as well as the future aspects of the study.

- How Casson parameter influences the flow profiles of Co–Au ~ paraffin hybrid nanofluid over the wedge surface?
- What is the impact of surface expansion/contraction on the momentum and thermal boundary layers of the Casson hybrid nanofluid?
- What are the relative contributions of thermal radiation and Joule heating in improving thermal energy transport within the hybrid nanofluid system?

*Importance of used nanoparticles* The choice of Co and Au nanoparticles is motivated by their distinct and complementary thermophysical properties which are crucial for the present investigation. The Co nanoparticles are known for higher thermal conductivity, strong magnetic responsiveness, making them suitable for magneto-thermal and electrically conducting flow applications. On the other hand, Au nanoparticles exhibit excellent thermal conductivity, higher chemical inertness, and also biocompatibility. Moreover, Au nanoparticles possess unique surface resonance properties which enhance energy transport at nanoscales and relevant for various biomedical systems.

## Mathematical formulation

A stable, laminar, 2D transportation of a Casson hybridized fluid over a permeable expanding or contracting wedge. The coordinate $x$ is measured along the wedge’s wall, while the $y$-axis is perpendicular to it.

- The angle of the wedge is $m$ $\left( {0 \le m \le 1} \right)$ with $m = 0\left( {\xi = 0} \right)$ characterizes the flow at a horizontal flat plate $\left( {\Omega = 0} \right)$ and $m = 1\left( {\xi = 1} \right)$ indicates the motion near the plane stagnation-point on a vertical flat plate $\left( {\Omega = \pi } \right)$.
- A magnetic field $B\left( x \right) = B_{0} x^{{\frac{{\left( {m - 1} \right)}}{2}}}$ is acting orthogonal to the wedge.
- The ambient velocity is $u_{e} \left( x \right) = U_{e} x^{m}$,while the stretching velocity of the wedge with a velocity $u_{w} \left( x \right) = U_{w} x^{m}$ where $U_{e}$ is a positive constant for the free stream, while $U_{w} > 0$ is demonstrating for the stretching wedge, $U_{w} < 0$ is indicating a shrinking wedge and $U_{w} = 0$ for a static wedge.
- Moreover,$m = \frac{\xi }{{\left( {2 - \xi } \right)}}$ represents the Hartree pressure gradient parameter, where $\xi$ corresponds to $\Omega = \xi \pi$ for the total angle of the wedge.
- The wall temperature is considered to be $T_{w}$ and the ambient temperature is to be $T_{\infty }$.

The constitutive rheological relation governing the Casson fluid may be expressed as follows

1 $$\tau_{ij} = \left\{ {\begin{array}{*{20}l} {2\left( {\mu_{d} + \frac{{p_{y} }}{{\sqrt {2\pi } }}} \right)e_{ij} ,} \hfill & {{\text{if }}\pi > \pi_{c} ,} \hfill \\ {2\left( {\mu_{d} + \frac{{p_{y} }}{{\sqrt {2\pi_{c} } }}} \right)e_{ij} ,} \hfill & {{\text{if }}\pi < \pi_{c} .} \hfill \\ \end{array} } \right.$$

where $e_{ij}$ designates the $(i,j)^{th}$ component of the rate of the strain tensor. The symbol $\pi_{c}$ signifies a threshold value of $\pi = e_{ij} e_{ij}$ in accordance with the non-Newtonian constitutive paradigm.

The standard equations with above assumptions are as follows (Izady et al.^22^):

2 $$\partial_{x} u + \partial_{y} v = 0,$$

3 $$u \partial_{x} u + v\partial_{y} u = u_{e} d_{x} u_{e} + \frac{{\mu_{hnf} }}{{\rho_{hnf} }}\left( {1 + \frac{1}{\beta }} \right)\partial_{yy} u - \frac{{\sigma_{hnf} }}{{\rho_{hnf} }}B^{2} \left( {u - u_{e} } \right),$$

4 $$u \partial_{x} T + v \partial_{y} T = \left[ {\frac{{k_{hnf} }}{{(\rho c_{p} )_{hnf} }} + \frac{{16\sigma_{0} T_{\infty }^{3} }}{{3k^{ * } (\rho c_{p} )_{hnf} }}} \right]\partial_{yy} T + \frac{1}{{(\rho c_{p} )_{hnf} }}\left( {\partial_{y} u} \right)^{2} + \frac{{\sigma_{hnf} B^{2} u^{2} }}{{(\rho c_{p} )_{hnf} }},$$

The corresponding surface constraints are reported as

5 $$\left. \begin{gathered} at\,\,y = 0:u = u_{w} ,\,\,\,v = v_{w} ,\,\,\,T = T_{w} , \hfill \\ as\,\,\,y \to \infty :u \to u_{e} ,\,\,\,\,T \to T_{\infty } \,.\,\,\,\,\,\,\,\,\,\,\,\,\,\,\,\,\,\,\, \hfill \\ \end{gathered} \right\}$$

Here, at the wedge surface, $y = 0$ the proposed condition denotes a moving and permeable wedge with prescribed wall temperature. Particularly, $u_{w} \left( x \right)$ represents the stretching/shrinking wedge surface while $v_{w} = \sqrt {\frac{{(m + 1)\upsilon_{f} U_{e} x^{m - 1} }}{2}} s$ accounts for wall mass transfer through suction/injection. These conditions are directly influence the momentum and thermal; boundary layers; wall stretching enhances fluid acceleration and reduces boundary layer thickness whereas suction stabilizes the flow and intensifies heat transfer while injection opposes it. However, boundary conditions are representative of real-world applications such as flow over aerodynamic wedges, cooling fins, and coated surfaces subjected to controlled heating and mass transfer.

The following are the similarity rules utilized for the transformation of the non-dimensional model:

6 $$\eta = \left( {\frac{{\left( {m + 1} \right)U_{e} }}{{2\upsilon_{f} }}} \right)^{\frac{1}{2}} x^{{\frac{m + 1}{2}}} y,\,\,\,\psi = \left( {\frac{{2\upsilon_{f} U_{e} }}{m + 1}} \right)^{\frac{1}{2}} x^{{\frac{m + 1}{2}}} f\left( \eta \right),u = \partial_{y} \psi ,v = - \partial_{x} \psi ,\,\,\theta \left( \eta \right) = \frac{{T - T_{\infty } }}{{T_{w} - T_{\infty } }},$$

In particular, the assumptions, $\psi$ is used as a stream function and $\eta$ is the similarity variable. The velocity components $u$ and $v$ satisfying the Cauchy criteria and $\theta$ is the transformation of the dimensional variable for the temperature.

Using the similarity mentioned above rules the model presented in Eqs. (3–5) are transformed to,

7 $$\left( {1 + \frac{1}{\beta }} \right)f^{\prime\prime\prime} + \frac{{\chi_{2} }}{{\chi_{1} }}\left[ {ff^{\prime\prime} + \frac{1}{m + 1}2m\left( {1 - f^{{\prime}{2}} } \right) + \frac{{\chi_{3} }}{{\chi_{2} }}\frac{1}{m + 1}2M\left( {1 - f^{\prime}} \right)} \right] = 0,$$

8 $$\frac{1}{\Pr }\left[ {\chi_{4} + \frac{4}{3}Rd} \right]\theta^{\prime\prime} + \chi_{5} f\theta^{\prime} + \chi_{1} Ecf^{{\prime\prime}{2}} + \chi_{3} MEcf^{{\prime}{2}} = 0$$

Considering the surface constraints

9 $$\left. \begin{gathered} f\left( 0 \right) = s,\,\,f^{\prime}\left( 0 \right) = \lambda ,\,\,\theta \left( 0 \right) = 1, \hfill \\ f^{\prime}\left( \infty \right) \to 1,\,\,\theta \left( \infty \right) \to 0 \hfill \\ \end{gathered} \right\}$$

The expressions for the dimensionless terms used in the flow phenomena are,

10 $$\left. {M = \frac{{\sigma_{f} B_{0}^{2} }}{{\rho_{f} U_{e} }},\,\,\,\,\lambda = \frac{{U_{w} }}{{U_{e} }},\Pr = \frac{{\upsilon_{f} }}{{\alpha_{f} }},\,\,Rd = \frac{{4\sigma_{0} T_{\infty }^{3} }}{{k_{f} k^{*} }}} \right\}$$

where for $\lambda > 0$ stretching wedge, for a shrinking wedge ($\lambda < 0$), $\lambda = 0$ for a static wedge.

The constants used in the above equations are

$$\chi_{1} = \frac{{\mu_{hnf} }}{{\mu_{f} }},\chi_{2} = \frac{{\rho_{hnf} }}{{\rho_{f} }},\chi_{3} = \frac{{\sigma_{hnf} }}{{\sigma_{f} }},\chi_{4} = \frac{{k_{hnf} }}{{k_{f} }},\chi_{5} = \frac{{(\rho c_{p} )_{hnf} }}{{(\rho c_{p} )_{f} }}.$$

Here, the thermophysical models for hybrid nanofluids are

$$\frac{{\mu_{hnf} }}{{\mu_{f} }} = \left[ {\frac{1}{{(1 - \phi_{1} )^{2.5} (1 - \phi_{2} )^{2.5} }}} \right],\,\frac{{\rho_{hnf} }}{{\rho_{f} }} = \left\{ {\left[ {1 - \phi_{1} + \phi_{1} \frac{{\rho_{s1} }}{{\rho_{f} }}} \right](1 - \phi_{2} ) + \frac{{\rho_{s2} }}{{\rho_{f} }}\phi_{2} } \right\},$$

$$\frac{{\sigma_{hnf} }}{{\sigma_{f} }} = \left\{ {1 + 3\frac{{\left( {\frac{{\phi_{1} \sigma_{1} + \phi_{2} \sigma_{2} }}{{\sigma_{f} }} - (\phi_{1} + \phi_{2} )} \right)}}{{2 + \left( {\frac{{\sigma_{1} \phi_{1} + \sigma_{2} \phi_{2} }}{{\sigma_{f} (\phi_{1} + \phi_{2} )}}} \right) - \left( {\frac{{\sigma_{1} \phi_{1} + \sigma_{2} \phi_{2} }}{{\sigma_{f} }} - (\phi_{1} + \phi_{2} )} \right)}}} \right\}$$,

$$\frac{{(\rho c_{p} )_{hnf} }}{{(\rho c_{p} )_{f} }} = \left\{ {(1 - \phi_{2} )\left[ {1 - \phi_{1} + \phi_{1} \frac{{(\rho c_{p} )_{s1} }}{{(\rho c_{p} )_{f} }}} \right] + \phi_{2} \frac{{(\rho c_{p} )_{s2} }}{{(\rho c_{p} )_{f} }}} \right\}$$,

$$\frac{{k_{hnf} }}{{k_{nf} }} = \left[ {\frac{{k_{s2} + 2k_{nf} - 2\phi_{2} (k_{nf} - k_{s2} )}}{{k_{s2} + 2k_{nf} + 2\phi_{2} (k_{nf} - k_{s2} )}}} \right], \, \,{\mathrm{where}}\,\frac{{k_{nf} }}{{k_{f} }} = \left[ {\frac{{k_{s1} + 2k_{f} - 2\phi_{1} (k_{f} - k_{s1} )}}{{k_{s1} + 2k_{f} + 2\phi_{1} (k_{f} - k_{s1} )}}} \right]$$

The primary physical parameters are the skin friction coefficient, $C_{f}$, and the local Nusselt number, $Nu_{x}$ which are described below:

11 $$C_{f} = \frac{{\tau_{w} }}{{\rho_{f} u_{e}^{2} }},$$

12 $$Nu_{x} = \frac{{xq_{w} }}{{k_{f} \left( {T_{w} - T_{\infty } } \right)}},$$

Specifically, $\tau_{w}$ denotes shear stress, while the heat flux, denoted by $q_{w}$, as shown below:

13 $$\tau_{w} = \mu_{hnf} \left( {\frac{\partial u}{{\partial y}}} \right)_{y = 0} ,$$

14 $$q_{w} = - k_{hnf} \left( {\frac{\partial T}{{\partial y}}} \right)_{y = 0} + \left( {\frac{ - 4}{3}\frac{{\sigma_{0} }}{{k^{*} }}\frac{{\partial T^{4} }}{\partial y}} \right)_{y = 0}$$

By applying the similarity transformations, we can derive

15 $$C_{f} \left[ {\frac{{2{\mathrm{Re}}_{x} }}{{\left( {m + 1} \right)}}} \right]^{\frac{1}{2}} = \frac{{\mu_{hnf} }}{{\mu_{f} }}f^{\prime\prime}\left( 0 \right),$$

16 $$\sqrt {\frac{2}{{\left( {m + 1} \right)}}} \times \left[ {{\mathrm{Re}}_{x} } \right]^{{\frac{ - 1}{2}}} Nu_{x} = - \left[ {\frac{{k_{hnf} }}{{k_{f} }} + \frac{4}{3}Rd} \right]\theta^{\prime}\left( 0 \right)$$

It should be noted that ${\mathrm{Re}}_{x} = \frac{{U_{e} x}}{{\upsilon_{f} }}$.

## Simulation of the problem

The transformed nonlinear ODEs are evaluated through numerical methods. Therefore, due to lack of initial conditions in handling the solution numerically, the utility of shooting technique is also vital. The reformulated first-order ODEs for the proposed mathematical model presented in Eqs. (7) and (8) are considered as:

$$\begin{gathered} f = y_{1} ,f^{\prime} = y_{2} ,f^{\prime\prime} = y_{3} , \hfill \\ f^{\prime\prime\prime} = - \frac{{\chi_{2} }}{{\chi_{1} }}\left[ {ff^{\prime\prime} + \frac{1}{m + 1}2m\left( {1 - f^{{\prime}{2}} } \right) + \frac{{\chi_{3} }}{{\chi_{2} }}\frac{1}{m + 1}2M\left( {1 - f^{\prime}} \right)} \right]/\left( {1 + \frac{1}{\beta }} \right), \hfill \\ \Rightarrow y_{3}^{\prime } = - \frac{{\chi_{2} }}{{\chi_{1} }}\left[ {y_{1} y_{3} + \frac{2m}{{m + 1}}\left( {1 - y_{2}^{2} } \right) + \frac{{\chi_{3} }}{{\chi_{2} }}\frac{2M}{{m + 1}}\left( {1 - y_{2} } \right)} \right]/\left( {1 + \frac{1}{\beta }} \right) \hfill \\ \end{gathered}$$

$$\begin{gathered} \theta = y_{4} ,\theta^{\prime} = y_{5} , \hfill \\ \theta^{\prime\prime} = - {{\left( {\chi_{5} f\theta^{\prime} + \chi_{1} Ecf^{{\prime\prime}{2}} + \chi_{3} MEcf^{{\prime}{2}} } \right)} \mathord{\left/ {\vphantom {{\left( {\chi_{5} f\theta^{\prime} + \chi_{1} Ecf^{{\prime\prime}{2}} + \chi_{3} MEcf^{{\prime}{2}} } \right)} {\frac{1}{\Pr }\left[ {\chi_{4} + \frac{4}{3}Rd} \right]}}} \right. \kern-0pt} {\frac{1}{\Pr }\left[ {\chi_{4} + \frac{4}{3}Rd} \right]}}, \hfill \\ \Rightarrow y_{5}^{\prime } = - {{\left( {\chi_{5} y_{1} y_{5} + \chi_{1} Ecy_{3}^{2} + \chi_{3} MEcy_{2}^{2} } \right)} \mathord{\left/ {\vphantom {{\left( {\chi_{5} y_{1} y_{5} + \chi_{1} Ecy_{3}^{2} + \chi_{3} MEcy_{2}^{2} } \right)} {\frac{1}{\Pr }\left[ {\chi_{4} + \frac{4}{3}Rd} \right]}}} \right. \kern-0pt} {\frac{1}{\Pr }\left[ {\chi_{4} + \frac{4}{3}Rd} \right]}} \hfill \\ \end{gathered}$$

With the surface conditions

$$y_{1} (0) = s,\,\,y_{2} (0) = \lambda ,\,\,y_{3} (0) = \alpha_{1} ,y_{4} (0) = 1,y_{5} (0) = \alpha_{2}$$

**Fig. 1:**
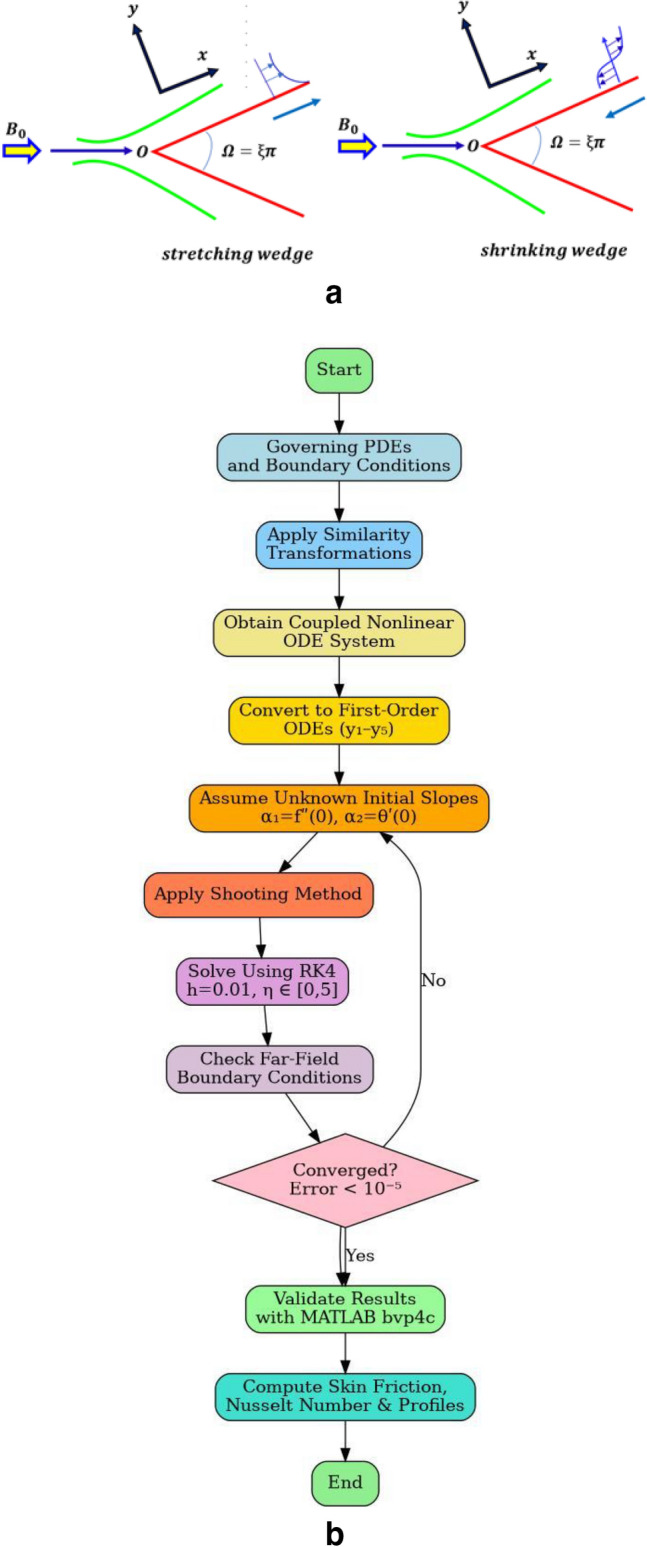
(**a**) Flow configuration describing the flow through stretching and shrinking wedge. (**b**) Flowchart of the numerical solution procedure for the proposed model.

The choice of these unknown assumptions of $\alpha_{1}$ and $\alpha_{2}$ are to be computed from the shooting technique validating the boundary conditions. Then traditional Runge–Kutta fourth-order is adopted to obtain the solution of the set of coupled ODEs with a proper choice of step length 0.01 within the range of $\eta \in [0,5]$ setting $\eta_{\infty } \to 5$. The iterative procedure is perpetuated until a convergence tolerance of 10^–5^ is attained for the computed profiles. However, the computation is carried out for the utilization of MATLAB software with built-in function bvp4c. Figure 1b illustrate the flowchart of the numerical solution procedure based on the shooting method.

## Validation of the result and discussion

The recent presentation deals with the movement of Casson-based hybridized nanomaterial associated with the integrating effect of the Cobalt (Co) and gold (Au) nanoparticles, considering the liquid paraffin oil which passed over a porous expanding/contracting wedge surface. The interpretation of magnetization alongside thermal radiation and magnetic dissipation urges to enhance the flow and heat transport phenomena. The model of thermophysical attributes of both the nanofluid and the hybrid nanofluids relating to the viscosity, density, conductivity, etc., is deployed within the text and the thermophysical attributes of both the nanoparticles and the base liquid paraffin are deployed in Table 1. The numerical analysis of the non-Newtonian fluid flow configuration, along with the influencing factors, is conducted after validating the present results against existing data. Table 2 displays the conformity of the current outcome with the result of Izady et al.^22^, considering the fixed values of the power index and keeping fixed the remaining presented in the caption. Both the numerical outcomes are coinciding, representing the validation as well as the convergence property of the working methodology.

**Table 1:** Thermophysical properties of Paraffin, cobalt, and gold (Yang et al.^23^).

Properties	Paraffin	Cobalt	Gold
$\rho \left( {{\mathrm{kg}}\,{\mathrm{m}}^{ - 3} } \right)$	802	8900	19,300
$C_{p} \left( {{\mathrm{J}}\,{\mathrm{kgK}}^{ - 1} } \right)$	2320	420	129.1
$k\left( {{\mathrm{W}}\,{\mathrm{mK}}^{{ - {1}}} } \right)$	0.23	100	320
$\sigma \left( {\Omega {\mathrm{m}}} \right)^{ - 1}$	$1.6 \times 10^{ - 14}$	$1.602 \times 10^{7}$	$4.5 \times 10^{7}$

**Table 2:** Comparison of $f^{\prime\prime}(0)$ for different values of $m$ at $M = Rd = s = \phi_{1} = \phi_{2} = \lambda = 0$.

$m$	Previous (Izady et al.^22^)	Present
0	0.4696	0.4696566
0.2	0.802125	0.8021255
0.5	1.038903	1.0389035

## Characterizing factors and their ranges

The fluid momentum and temperature patterns are obtained, considering various characteristic factors and their specific ranges as gathered from different literature. In general, the impact of magnetization $\left( M \right)$ affecting the flow phenomena with the power index $\left( m \right)$ parameter is considered as $0 \le M \le 4$ and $0.2 \le m \le 1$ respectively. The non-Newtonian properties of the Casson parameter $(\beta )$ is deployed for both the higher and lower values, indicating the Newtonian and the non-Newtonian behaviour on the flow dynamics and the variation is organized within the numerical range of $1 \le \beta \le 3.$ The velocity stretching ratio factor $(\lambda )$ on the flow behaviour is constructed and the numerical range is deployed considering $0 \le \lambda \le 0.6$. The surface permeability demonstrates the effect of suction and injection $\left( {s > 0} \right)/\left( {s < 0} \right)$ on the flow profile, with the range starting from $- 0.5 \le s \le 0.5.$ The rate of heat transmission is influenced by parameters such as thermal radiation $\left( {Rd} \right)$ and the Eckert number $\left( {Ec} \right)$ with ranges set at $0 \le Rd \le 2$ and $1 \le Ec \le 3$, respectively. Moreover, the analysis of several factors also distresses the shear rate presenting the skin friction coefficients and the heat transportation efficiency such as Nusselt number significantly those are deployed through graphs. Particularly, each of the profiles is reported for a comparative study considering Cobalt ~ Paraffin nanofluid and Cobalt + gold ~ Paraffin hybrid nanofluid.

## Analysis of various factors

**Fig. 2:**
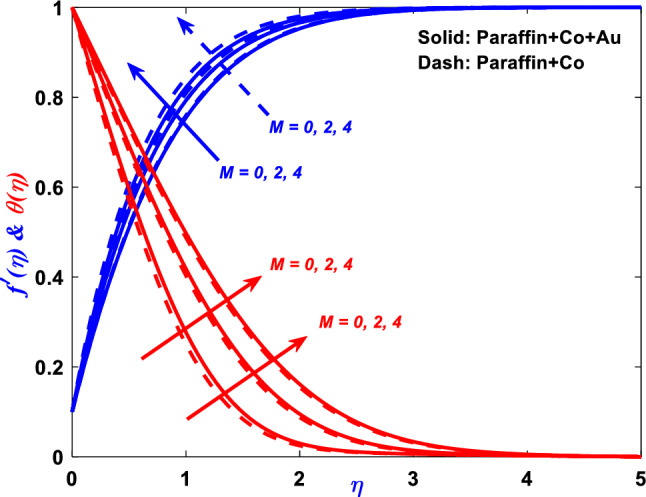
Behavior of magnetic parameter on velocity and temperature distributions.

**Fig. 3:**
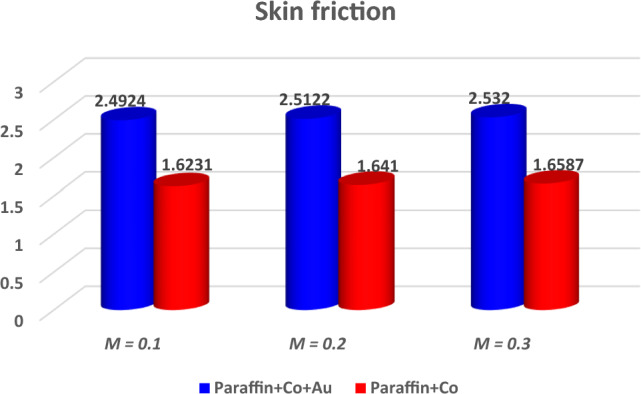
Numerical behaviour of magnetic parameter on skin friction.

**Fig. 4:**
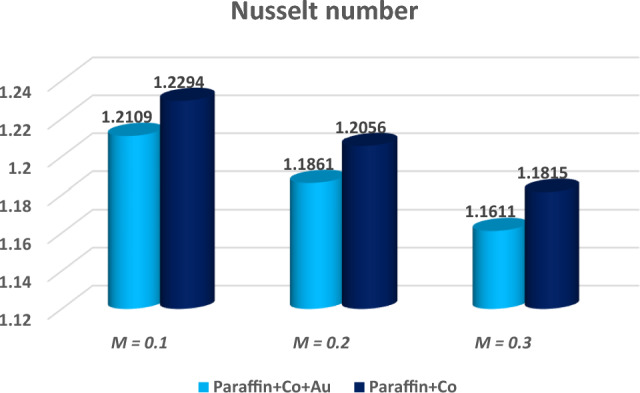
Numerical behaviour of magnetic parameter on Nusselt number.

The graphical illustration of the momentum along with the energy distribution of fluid are reported for the variation of different terms affecting the profiles. The applied magnetism that suggests the important properties of MHD affecting the fluid motion and the temperature is displayed in Fig. 2 within the aforementioned range. The proposed numerical value of $M$, specifically $M = 0$ represents the profile’s behaviour in the absence of magnetization effects, while an increasing nonzero variation highlights the influence of magnetization. The generative resistive force i.e., the Lorentz force for the inclusion of the applied magnetism deliberately resists the fluid motion toward the surface region and this leads to a significant declination in the fluid motion. This behaviour of the magnetization also leads to the diminishing of the velocity bounding surface and the results are irrespective of the consideration of the along with hybrid. The addition of Cobalt nanoparticles into the Paraffin performing the nanofluid shows greater density as well as the thermal efficiency of the nanofluid. The enhanced conductivity overrides the fact of density which significantly enhances the fluid velocity. Further, the inclusion of additional gold nanoparticles into the nanofluid performing the behaviour of hybrid is more influential with greater conductivity and the fluid velocity overrides the role of nanofluid at all points. The thinning of the bounding surface resulting from increased magnetization promotes energy accumulation in the fluid’s lower region due to particle deposition, leading to a rise in thermal energy. As an output, a notable upsurge in the fluid temperature is obtained within the entire region and this behaviour is rendered for the case of any fluid considered. Particularly, the flow phenomenon of the temperature profile is more effective in the situation of a hybridized fluid rather than the behaviour of a single nanofluid. The diversification of the magnetization shows its important role in the shear rate profile which is deployed in Fig. 3. This suggests that magnetization influences the skin friction coefficient. The findings indicate that stronger magnetization, which suppresses fluid velocity, leads to an increased velocity gradient, thereby enhancing the skin friction profile at the surface. Again, the influence is greater in the hybrid situation than the impact on the nanofluid and this fact is rendered by the increasing conductivity of the hybridized fluid. The rate of Nusselt number is also affected by the contribution of the magnetization and the variation is presented for the different cases. The illustration reveals that with the enhanced magnetization the heat transport efficiency is significantly controlled in both the case of the fluid considered. The comparative analysis shows a notable enhancement in the rate presented by the Cobalt ~ Paraffin nanofluid than that of the Cobalt + Gold ~ Paraffin hybrid nanofluid (Fig. 4).

**Fig. 5:**
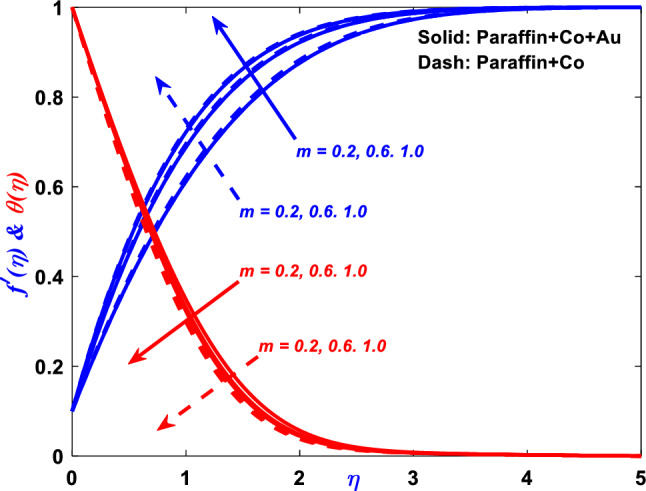
Behavior of power index on velocity and temperature distributions.

**Fig. 6:**
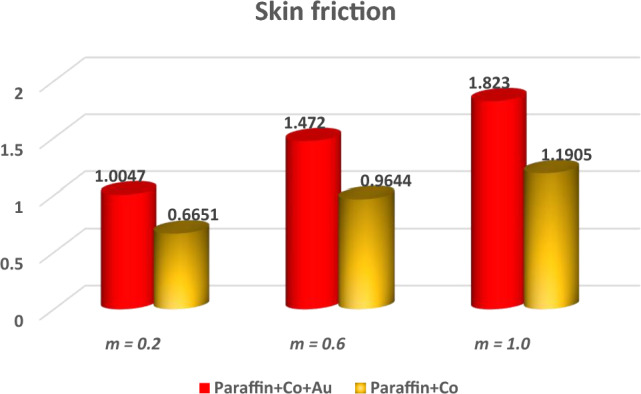
Numerical behaviour of power index on skin friction.

**Fig. 7:**
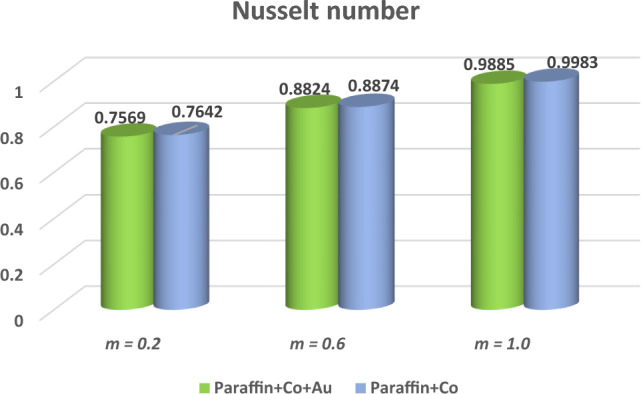
Numerical behaviour of power index on Nusselt number.

**Fig. 8:**
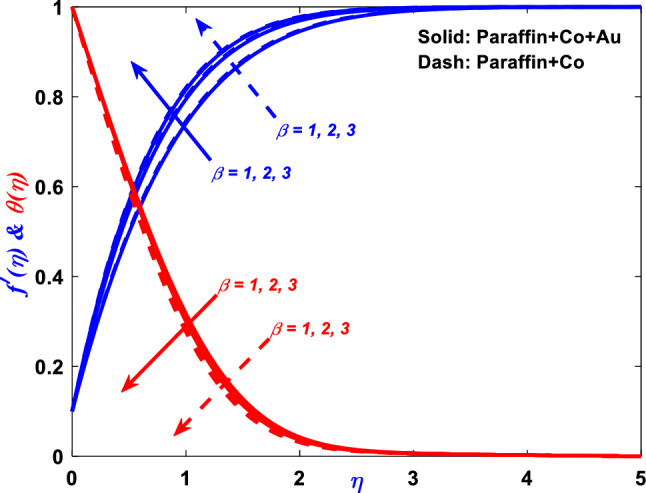
Behavior of Casson parameter on velocity and temperature distributions.

**Fig. 9:**
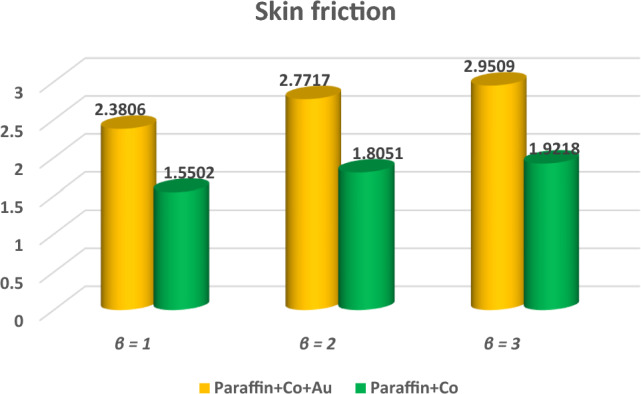
Numerical behaviour of Casson parameter on skin friction.

**Fig. 10:**
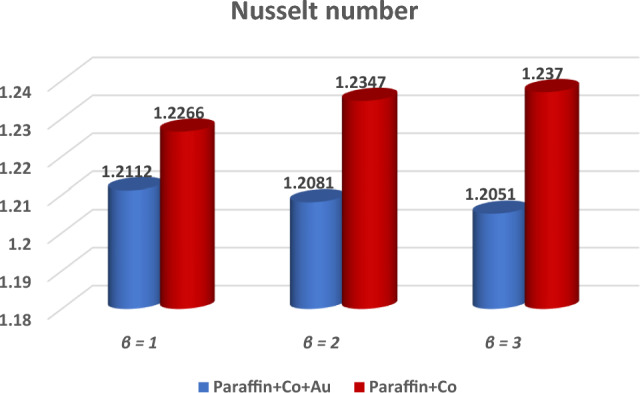
Numerical behaviour of Casson parameter on Nusselt number.

Figure 5 deploys the power-law index and its influence on the fluid momentum vis-a-vis temperature distribution. The evaluation is presented by comparing the behaviour of nanofluid with a hybrid. The contribution of the factor $m$ such as within the range of the behaviour is characterized in three distinct folds. In general, $m \le 1$ signifies pseudo-plastic fluids in which for the increasing shear rate the viscosity retards. Further, for $m = 1,$ the fluid behaviour becomes Newtonian and $m > 1$ shows dilatant fluid in which enhanced shear rate increases fluid viscosity. For shear-thinning behaviour with m is $m = 0.2,$ the viscosity decreases near the surface due to strong shear effects, resulting in a more pronounced velocity peak. In the case of Newtonian fluid, $m = 1,$ the momentum pattern is more uniform and the peak reduces in comparison to $m \le 1$ showing a thinning in the velocity-bounding surface. As mentioned earlier, the enhanced heat transfer capability of the hybridized fluid, compared to the conventional nanofluid, leads to a notable increase in fluid velocity throughout the entire domain. The thermal state of fluid is also affected by the power index and the lower value $m, \, m = 0.2,$ in the case of shear-thinning fluid, the reduced viscosity enhances convection which favours enhanced temperature distribution at all points. Further, with increasing variation of the factor m, the profile ceases to retard it significantly. The thermal bounding surface thickness increasingly decreases. This retardation is irrespective of the variation of the fluid considered here for the comparison. The insertion of gold nanoparticles into the nanofluid notably upsurges the temperature distribution, with the hybrid nanofluid exhibiting superior thermal performance due to its higher energy efficiency. The significant role of the factor $m$ is presented on the skin friction coefficient and the results are depicted in Fig. 6. As discussed, the lower m indicates a higher shear rate which means the viscosity decreases more significantly with increasing shear rate. This results in lower skin friction due to the resistance to the flow near the surface. Further, increasing *m* the profile increases significantly and this enhancement is more effective for the consideration of hybrid nanofluid which dominates the situation of nanofluid in each variation. The implication of the index factor on the pattern of the Nusselt number affecting the heat efficiency rate is presented in Fig. 7. As the shear rate increases, the shear-thinning behaviour leads to a reduction in viscosity, which improves convective heat transfer. This results in a significant enhancement in heat transfer efficiency as the fluid’s shear-thinning properties intensify. A slight rise in the velocity profile is noted for both the nanofluid and hybrid nanofluid, suggesting that the inclusion of gold nanoparticles upsurges the thermal efficiency of the hybrid nanofluid more effectively than the Co ~ Paraffin nanofluid. The flow attributes of the non-Newtonian fluid is exhibited by the variation of the Casson parameter influencing the momentum transport and thermodynamic condition of the fluid is portrayed in Fig. 8. The outcomes are illustrated for the consideration of both fluids. The Casson parameter is inversely related to the yield stress of the fluid which shows that the higher beta leads to low yield stress. These favours allow more fluid to flow. Small $\beta$ indicates high yield stress restricting the fluid to flow close to the surface and large $\beta$ indicates a low yield stress focuses on less resistance to flow. The enhanced particle concentrations such as the insertion of gold nanoparticles in the Co ~ paraffin nanofluid exhibiting the hybrid nanofluid synergistically elevates the momentum pattern but the thickness of the profile retards significantly. Further, high-yield stress controls convection that dominates over the conduction. This results in a thicker thermal bounding surface thickness and higher temperature within the domain is reflected. The superior conductivity properties of Co + Au ~ paraffin exhibit greater heat transfer in comparison to the Co ~ paraffin nanofluid. Figure 9 depicts the characteristic of the Casson parameter disturbing the shear rate**.** A discernible augmentation in the velocity gradient is observed, thereby intensifying the skin-friction coefficient across all spatial locations within the domain irrespective of the use of different fluids. However, the hybrid is more effective than the case of nanofluid considered in this discussion due to its advanced thermal properties. The Nusselt number is also precious by the non-Newtonian Casson parameter and the results are represented in Fig. 10. The thermal gradient favours enhancing the rate with the increasing Casson parameter and this enhancement is more effectively controlled by the consideration of hybrid nanofluid rather than the Co ~ paraffin nanofluid.

*Applications* The non-Newtonian Casson parameter combining Co and Au in paraffin shows important applications in aircraft technology. The aircraft engine cooling systems require effective heat transfer to manage high temperatures. The Casson parameter characterizes non-Newtonian behaviour, meaning the fluid remains stationary until the applied stress exceeds a specific yield value. The operation of aircraft fuel systems with varying pressures and temperature requires stable flow which is characterized by the Casson parameter.

**Fig. 11:**
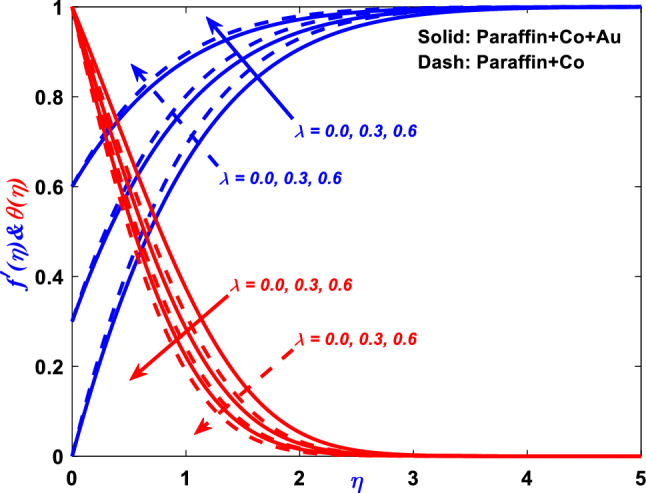
Evaluation of stretching ratio parameter on velocity and temperature.

**Fig. 12:**
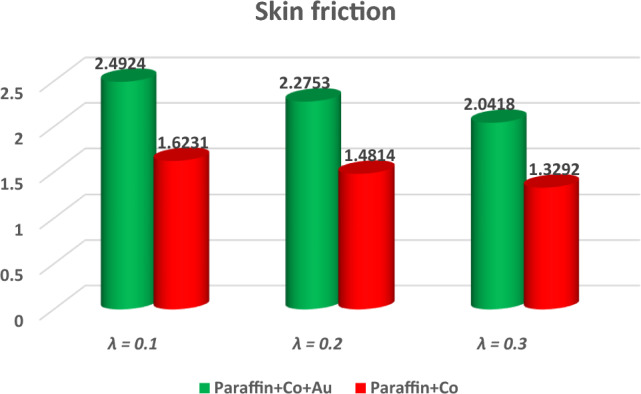
Numerical behaviour of stretching ratio parameter on skin friction.

**Fig. 13:**
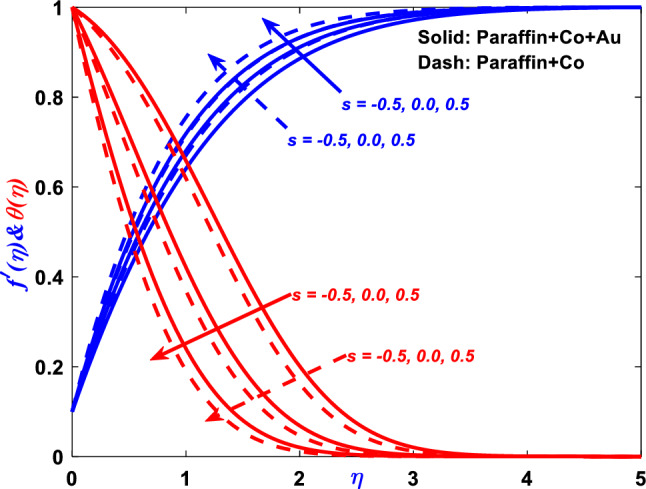
Behavior of suction/injection on velocity and temperature distributions.

**Fig. 14:**
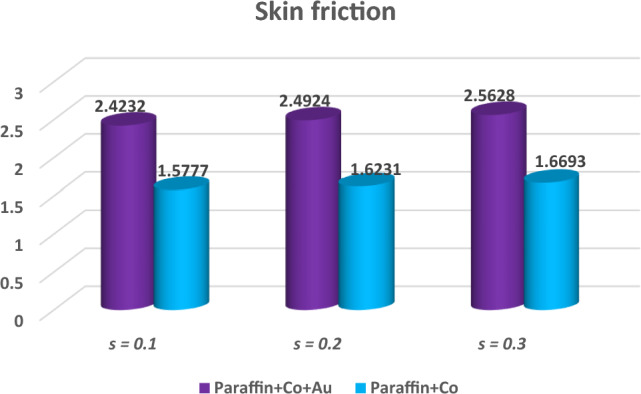
Numerical behaviour of suction/injection on skin friction.

**Fig. 15:**
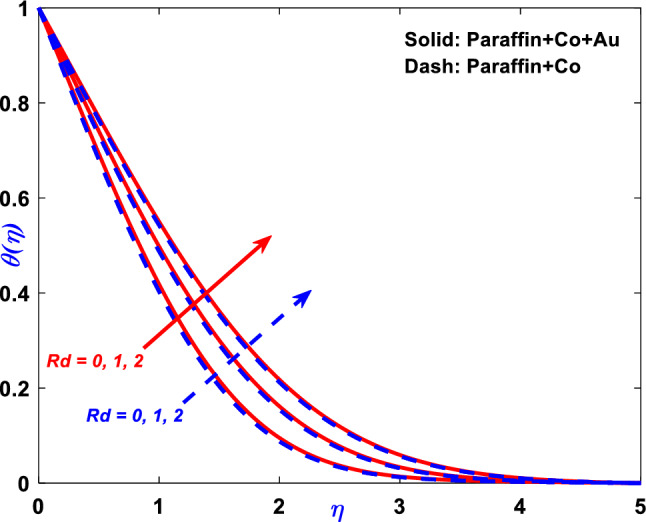
Behavior of thermal radiation on temperature distributions.

**Fig. 16:**
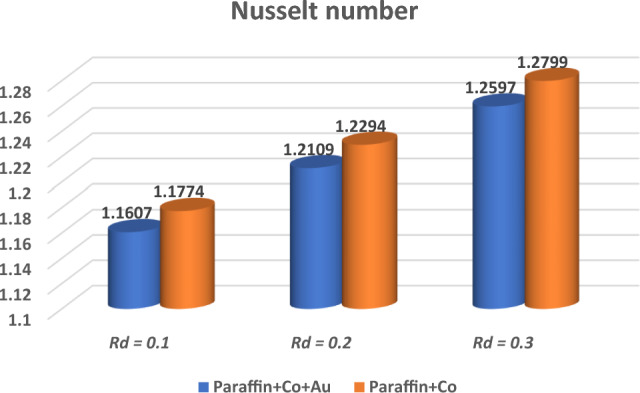
Numerical behaviour of thermal radiation on Nusselt number.

Figure 11 illustrates the significant influence of the extending ratio term on fluid motion and temperature distribution, providing a comparative analysis of the nano and hybridized fluid. For the increasing stretching parameter, the surface stretching becomes more pronounced and this causes a noteworthy heightening in the fluid motion along the direction of stretching. This leads to a declination in momentum near the bounding surface, exhibiting a similar pattern for both nanofluids and hybrid nanofluids. The single particle such as the interpretation of Co nanoparticles in Paraffin, amplifies the fluid velocity due to increasing momentum transfer by the cobalt nanoparticle. Further, the inclusion of the gold nanoparticles improves the thermal properties that lower the resistance, which enhances the thickness of the distribution. Also, increasing $\lambda$ leads to higher stretching that reduces the thermal bonding surface thickness. This enhances the heat transfer, reflecting lower fluid temperature away from the surface. The thermal conductivity of the Co nanoparticles improves the heat transfer, but is dominated by the hybrid nanofluid. The incorporation of gold nanoparticles improves thermal conductivity, leading to enhanced thermal properties and an overall increase in fluid temperature throughout the domain. The profile’s asymptotic behaviour fails to meet the boundary condition defined in the proposed model. In a concluding remark, it is seen that no stretching $(\lambda = 0)$ lead to a thicker thermal boundary layer, that leads to enhancing the fluid temperature, but increasing $\lambda$ retards it significantly. Figure 12 elaborates on the notable attribute of the factor on the rate of shear stress in two distinct cases. The results indicate a notable reduction in the velocity gradient, leading to a declination in skin friction across the entire surface. Further, this retardation is obtained for both nano and hybrid cases but the scenario of hybrid deploys greater behaviour than the nanofluid. The permeability of the elastic interface that reflects the properties of the suction/injection affecting the fluid velocity and energy layer displayed in Fig. 13. The negative values represent the injection scenario, where extra fluid in the surface region moves away, while the positive variation ($s, \, s = 0.5$) signifies the introduction of additional fluid into the system. Further, the assigned numerical values signify the transportation via impermeable surfaces. The fluid velocity rises steadily with increased suction, resulting in a thinner layer, while injection causes the opposite effect. As suction intensifies, the thermal boundary layer becomes thinner, whereas higher injection enhances the temperature profile. The comparative results presented for the variation of nanofluid and the hybrid reveal a greater thickness in both the velocity and temperature due to the inclusion of more nanofluids. Figure 14 shows a comparative analysis of the skin friction concerning the suction parameter. The result reveals that with the gradual increase of suction, the profile of the skin friction increases, and this increase is insignificant. Suction reduces the fluid velocity but it is counterproductive in case of velocity gradient. Thus, the suction induces a change in velocity gradient, and that enhances the skin friction. The addition of nanoparticles improves the thermal and momentum transfer that dominates the skin friction in both the case of nanofluid and hybrid nanofluid. The temperature distribution of the Casson fluid is affected by the factor thermal radiation which is presented in Fig. 15. The impact of the factor $Rd$ arises for the postulation of the heat flux from the Rosseland approximation. The assigned numerical value of $Rd$, $Rd = 0$ indicates the absence of thermal radiation whereas $Rd > 0$ shows the variation and its behaviour affecting the fluid temperature. For $Rd = 0$, the energy transfer is dominated by the conduction and convection mechanism which lead to a decelerating effect on the flow profile. An intensification in thermal radiation enhances energy transfer, leading to a rise in fluid’s energy. The inclusion of Co and Au nanoparticles enhances the thermal conductivity of the hybrid nanofluid, leading to a rise in temperature and a notable increase in the thermal flow pattern. Figure 16 elucidates the interpretation of thermal radiation on heat transfer efficiency, resulting in a notable growth of the rate of thermal transportation and a considerable increase in the Nusselt number. In both the figures the comparative evaluation reports that with superior thermal attributes of the hybridized fluids, the heat transportation efficiency is also favourable in increasing rather than the lower conductivity of nanofluid.

*Applications* Thermal radiation exerts a substantial influence in radiative heat transfer compared to conduction and convection, making it essential for aircraft management applications. The Casson hybrid nanofluid is used in thermal dissipation system where thermal radiation plays a critical role in heat deposition. The integration of Co and Au into Paraffin improves heat transfer due to their high conductivity, while the thermal radiation parameter regulates heat flux within the engine cooling system.

**Fig. 17:**
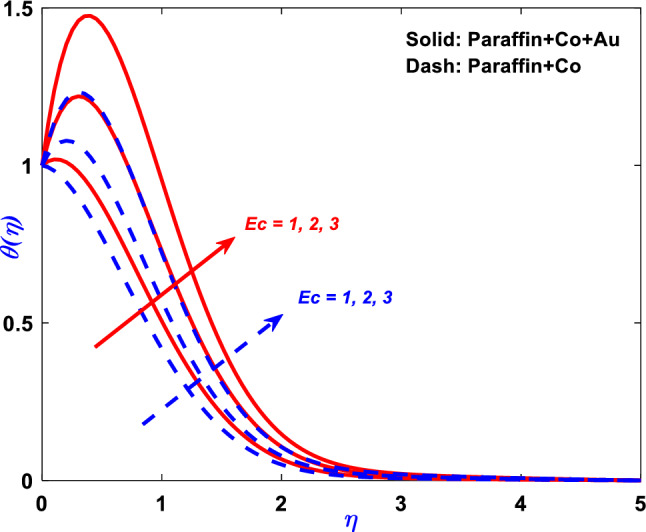
Behavior of Eckert number on temperature.

**Fig. 18:**
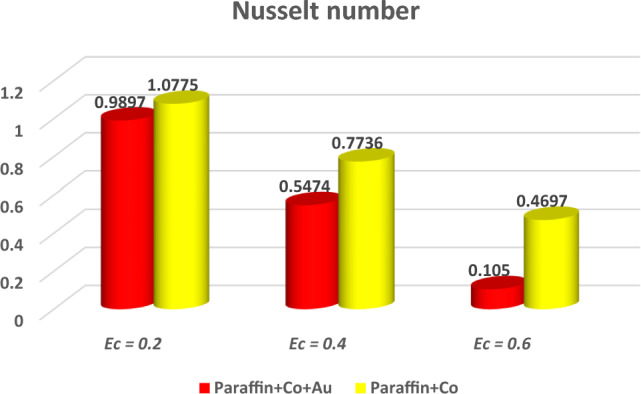
Numerical behaviour of Eckert number on Nusselt number.

Enthalpy significantly influences the fluid temperature and heat transfer rate. Its impact is analyzed using the Eckert number, which markedly modulates the fluid temperature and the Nusselt number, as illustrated in Figs. 17 and 18. Both the profiles are depicted for the consideration of Co ~ Paraffin nanofluid and Co + Au ~ Paraffin hybrid nanofluid which reveals a comparative study. The factor *Ec* primarily defined as the ratio of kinetic energy to the enthalpy which causes due to the integration of dissipative heat in the energy transport mechanism. Lower $Ec, \, Ec = 1$ presents minimal effects of viscous dissipation and the fluid’s energy remains uniform and slowing down the profile. Further increasing Eckert number, which gives rise to an increasing kinetic energy vis-à-vis retardation in the enthalpy, leads to a greater hike in the fluid temperature closer to the surface and gradually the profile retards to meet the corresponding boundary conditions. The results of the inclusion of additional gold nanoparticles in the Co ~ Parafin nanofluid, exhibiting the role of hybrid nanofluid, experience a greater conductivity in the thermal properties, leading to enhanced heat transfer phenomenon, and the fluid temperature significantly increases in the entire region, dominating the role of nanofluid. Further, the heat transfer efficiency is controlled by the increasing Eckert number, and this causes a retardation in the thermal transportation. The inclusion of more nanoparticles also favours controlling the heat transportation efficiency rather than the nanofluid situation.

## Conclusions

The transportation of Casson hybrid nanofluid exhibiting the role of Cobalt and Gold nanoparticles in the base liquid Paraffin over an expanding/contracting surface is deployed in this study. The additional interpretation of thermal properties is assessed by integrating thermal radiation, and Joule dissipation is also reported. The flow profiles are notably affected by surface permeability, and the numerical results presented for comparison successfully confirm the accuracy of this analysis. Moreover, the performance of distinct factors affecting the flow phenomena is deployed, and the important findings are described below;

- The numerical results demonstrate the validation of the proposed methodology by comparing it with existing literature, confirming its convergence.
- The inclusion of the Casson parameter in the fluid model enhances momentum while notably reducing the profile thickness due to its non-Newtonian characteristics.
- The Lorentz force causes a resistive effect that slows down the fluid motion.
- The permeability of the surface causes a decrease in the bounding surface thickness for both velocity and temperature distributions.
- The inclusion of gold nanoparticles in the Paraffin-based cobalt nanofluid exhibits greater strength and enhanced velocity as well as fluid temperature.
- The thermal transport properties, along with the heat transfer rate, are enhanced significantly with the increasing thermal radiation irrespective of the disparity of the nanofluid and hybrid nanofluid.
- The combination of dissipative heat and the Eckert number enhances control over the heat transfer rate in both fluids under consideration.

The present investigation reports neither the analysis of the physical significance of various factors but also utilized them in several applications. Future research can be directed toward analyzing tri-hybrid Casson nanofluids by incorporating an additional nanoparticle into the Cobalt-Gold-Paraffin base mixture. The present study can be extended by integrating the melting phenomenon to simulate more realistic de-icing and anti-icing scenarios. Implementation of an ANN could optimize the prediction of Nusselt number, skin friction, etc. A parametric study involving time-varying magnetic fields, nonlinear radiation can provide better significance in heat transport phenomena.

**Important notes: Despite of superior thermal conductivity and optical properties of gold nanoparticles, they are prohibitively expensive, which limits their viability for large-scale aerospace or industrial systems. Therefore, in alternative, Oxide-based nanoparticles, i.e., Al_2_O_3_, CuO, and carbon-based nanostructures, offer strong thermal conductivity improvements at a fraction of the cost.

## Data availability statement

The data that support the findings of this study are available from the corresponding author upon reasonable request.

## List of symbols:

$$u,\,v$$: Velocity components, $$\,{\mathrm{ms}}^{ - 1}$$
$B_{0}$: Uniform magnetic field
$$k$$: Thermal conductivity, $$\,{\mathrm{Wm}}^{ - 1} {\mathrm{K}}^{ - 1}$$
$$M$$: Magnetic parameter
${\mathrm{Re}}_{x}$: Local Reynolds number
$$\Pr$$: Prandtl number
$q_{w}$: Surface heat flux, $\,{\mathrm{Wm}}^{ - 2}$
$$T_{\infty }$$: Ambient temperature, K
$$\eta$$: Similarity variable
$$\theta \left( \eta \right)$$: Temperature distribution
$\lambda$: Buoyancy convection parameter
$$v$$: Kinematic viscosity, $${\mathrm{m}}^{2} {\mathrm{s}}^{ - 1} \,$$
$$\mu$$: Dynamic viscosity, $${\mathrm{kgm}}^{ - 1} {\mathrm{s}}^{ - 1}$$
$$\sigma$$: Electrical conductivity, $$\left( {\Omega {\mathrm{m}}} \right)^{ - 1}$$
$$x,\,y$$: Cartesian coordinates, m
$C_{f}$: Skin friction parameter
$$R$$: Radiative parameter
$Nu_{x}$: Local Nusselt number
$$s$$: Suction/injection
$T$: Temperature of the fluid, K
$$T_{w}$$: Surface temperature, K
$\xi$: Pressure gradient term
$\rho$: Fluid density, kgm^-3^
$$\psi$$: Stream function, m^2^s^-1^
$$\infty$$: Ambient condition
$u_{e} \left( x \right)$: Free stream velocity, ms^-1^
$\tau_{w}$: Wall shear stress, kgm^-1^s^2^
$$\beta$$: Casson parameter
